# Supplementary material for: Earth system models underestimate carbon fixation by plants in the high latitudes
Source: Nat Commun. 2019 Feb 21;10:885. doi: 10.1038/s41467-019-08633-z (PMC6385346; doi:10.1038/s41467-019-08633-z)
Supplement: Supplementary file 1 — Supplementary Information [file 41467_2019_8633_MOESM1_ESM.pdf]

Earth System Models Underestimate Carbon Fixation by Plants in the High Latitudes
- Supplementary Information -

Winkler et al.

Corresponding author: Alexander J. Winkler (alexander.winkler@mpimet.mpg.de)

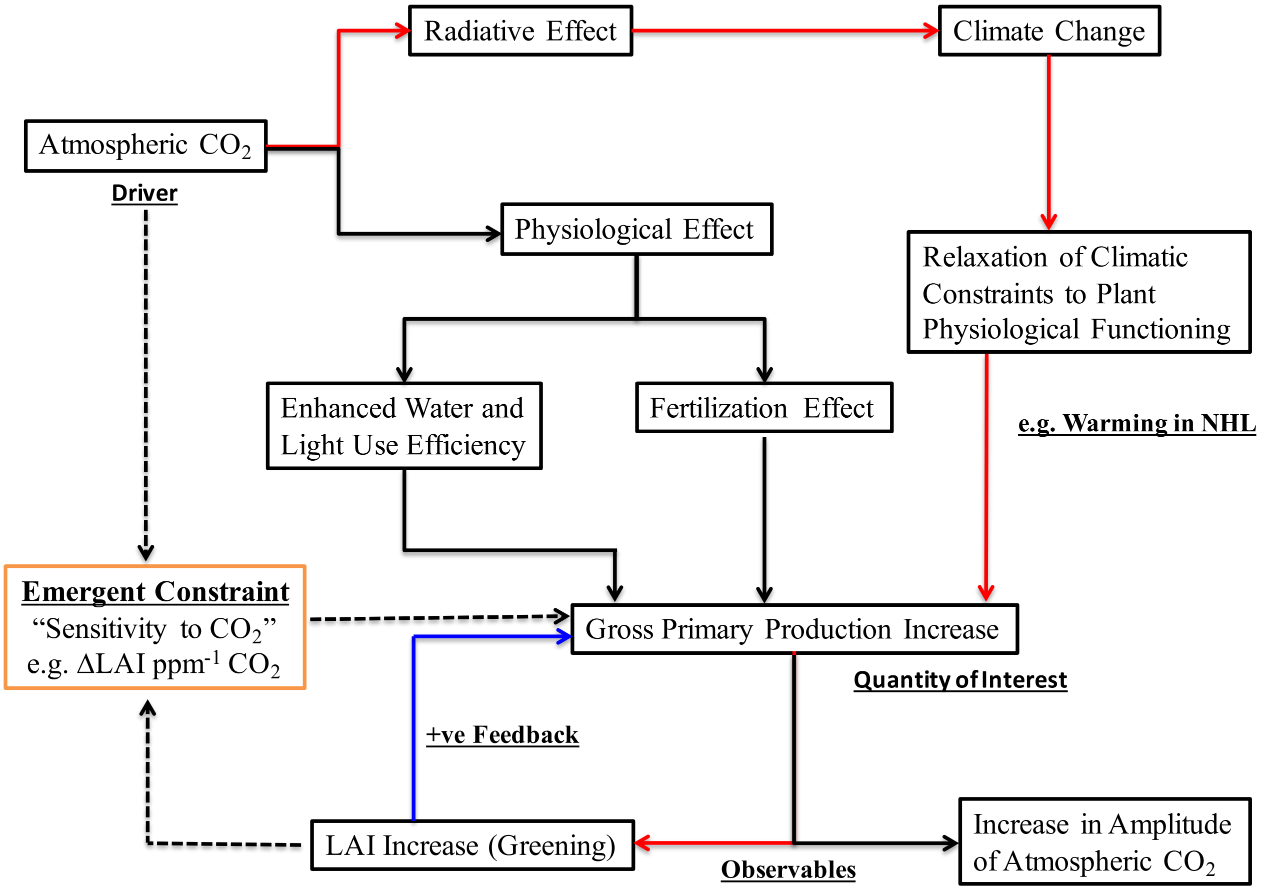

Supplementary Figure 1 | Schematic of the Emergent Constraint concept. The radiative and physiological effects of increasing atmospheric CO_2 concentration, in the range 280 to 560 ppm, are thought to increase GPP[2,3,12-14]. This is indirectly observed as changes in LAI[18,19] or the amplitude of the seasonal cycle of atmospheric CO_2[16,17,23]. The sensitivity of changes in observables to historical increase in CO_2 concentration (e.g., 280 to 400 ppm) can be thought of as an Emergent Constraint on model-projected changes in carbon cycle quantities (e.g., ΔGPP for CO_2 change from 280 to 560 ppm), if the inter-model variation of projections is linear, or nearly so, with respect to modelled historical sensitivities[23]. GPP enhancement due to the radiative effect (red arrows) was not included in Wenzel et al.[23] because their focus was on obtaining a constrained estimate for the physiological effect only. GPP enhancement from the positive feedback effect (blue arrow) is thought to be small[41] relative to the physiological and radiative effects, and included in our study.

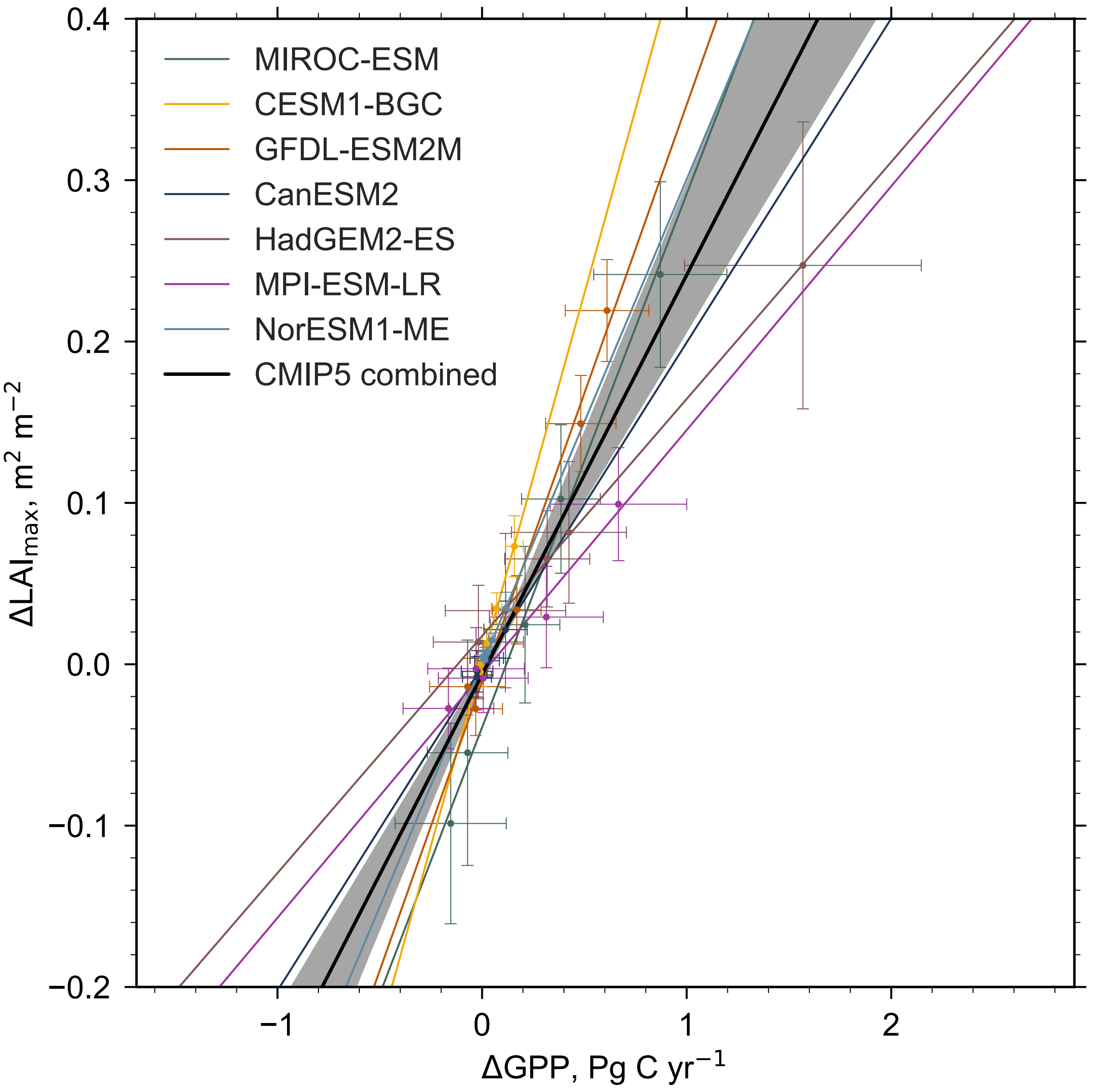

Supplementary Figure 2 | Linear relationship between concurrent changes in LAI$_{max}$ and annual mean GPP. Comparison of changes in LAI$_{max}$ and annual mean GPP for the historical period (1860 to 2005) for the NHL (60° N - 90° N) in the CMIP5 ensemble. The colored dots show values for 30 year chunks of the total time series (error bars denote one standard deviation). The colored lines represent the best linear fit for each model, while the black line indicates the best linear fit for all models. The 68% confidence interval estimated by bootstrapping is shown by the grey shading.

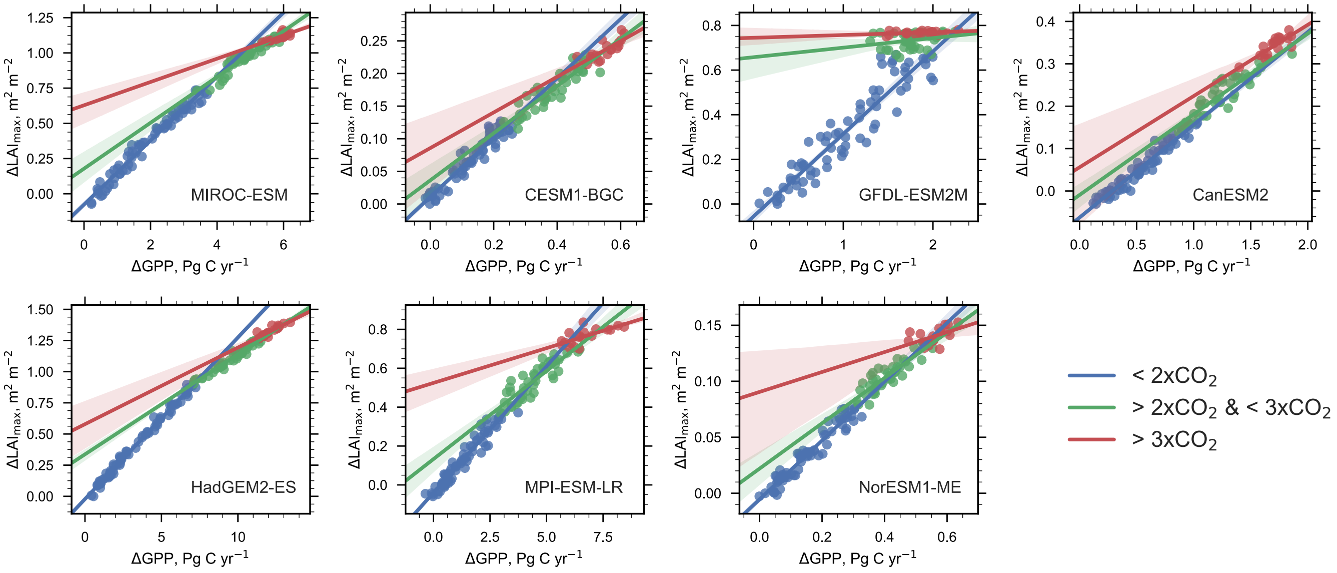

Supplementary Figure 3 | Relationship between ΔLAI_{max} and ΔGPP with increasing CO_2 forcing, starting from a pre-industrial concentration of 280 ppm (1xCO_2) to 4xCO_2. Blue colored dots represent the relation for concentration below 2xCO_2. Green colored dots between 2xCO_2 and 3xCO_2. Red colored dots between 3xCO_2 and 4xCO_2 (Supplementary Table 3). The respective colored lines are the regressions through those dots and the shading represents the 95% confidence interval.

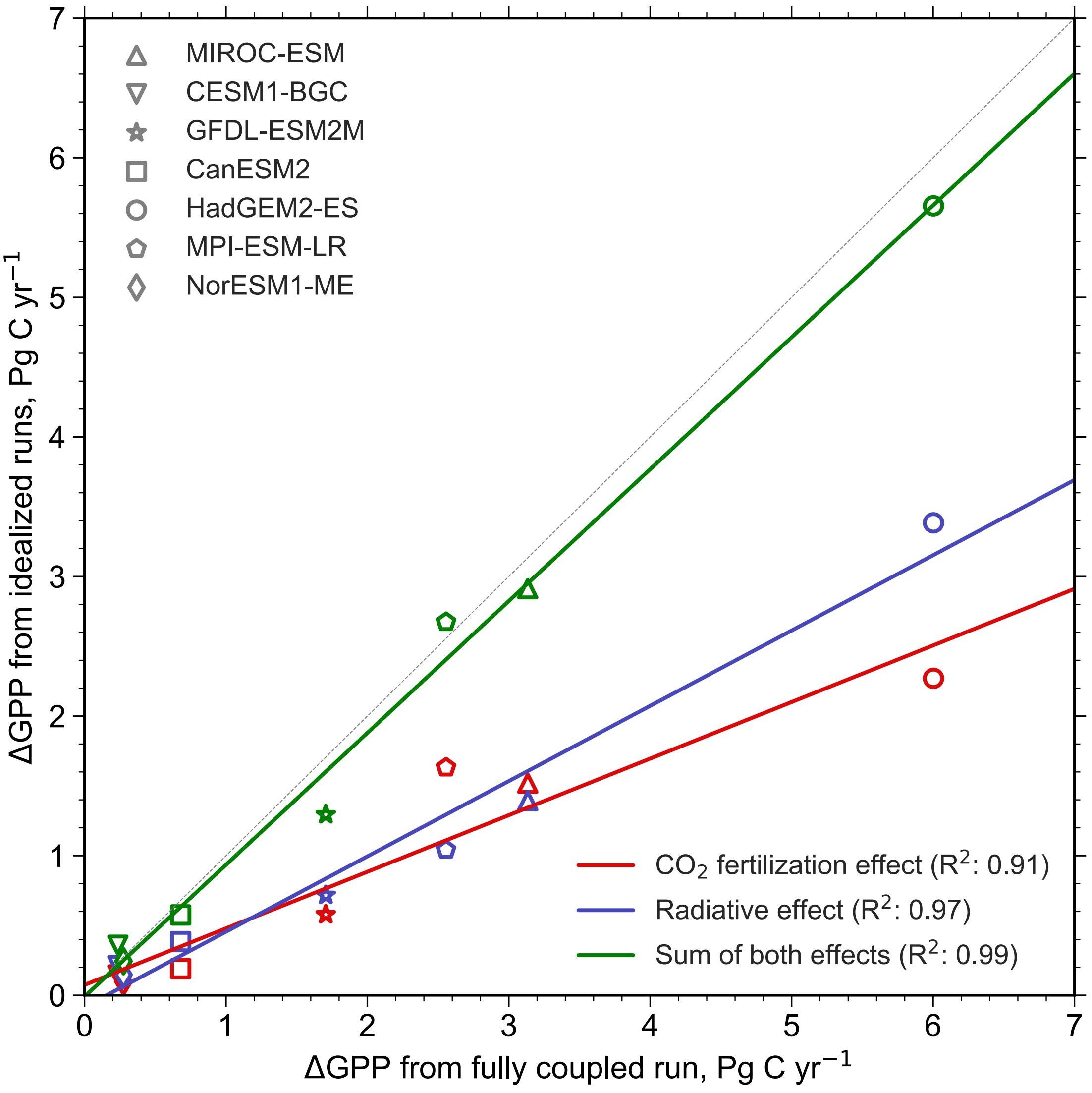

Supplementary Figure 4 | Radiative and fertilization effects on NHL photosynthesis. Changes in annual mean GPP for doubling of pre-industrial CO_2 concentration from 1%CO_2 (CO_2 concentration increased by 1% yr^{-1} from preindustrial level) fully coupled (radiative plus fertilization) CMIP5 model runs (*x*-axis) plotted against idealized simulations (*y*-axis) with only the radiative (CMIP5's esmFdbk)[6] or the fertilization (CMIP5's esmFixClim)[6] effect included runs. The markers show the corresponding model values from these runs. The colored lines are best fits to the respective markers (red for fertilization effect only, blue for radiative effect only). The green line represents the best fit to the sum of quantities from the idealized runs and the fully coupled run. Wenzel et al.[23] provided a constrained estimate for GPP projection considering the fertilization effect only. Including the radiative effect would at least double their estimate.

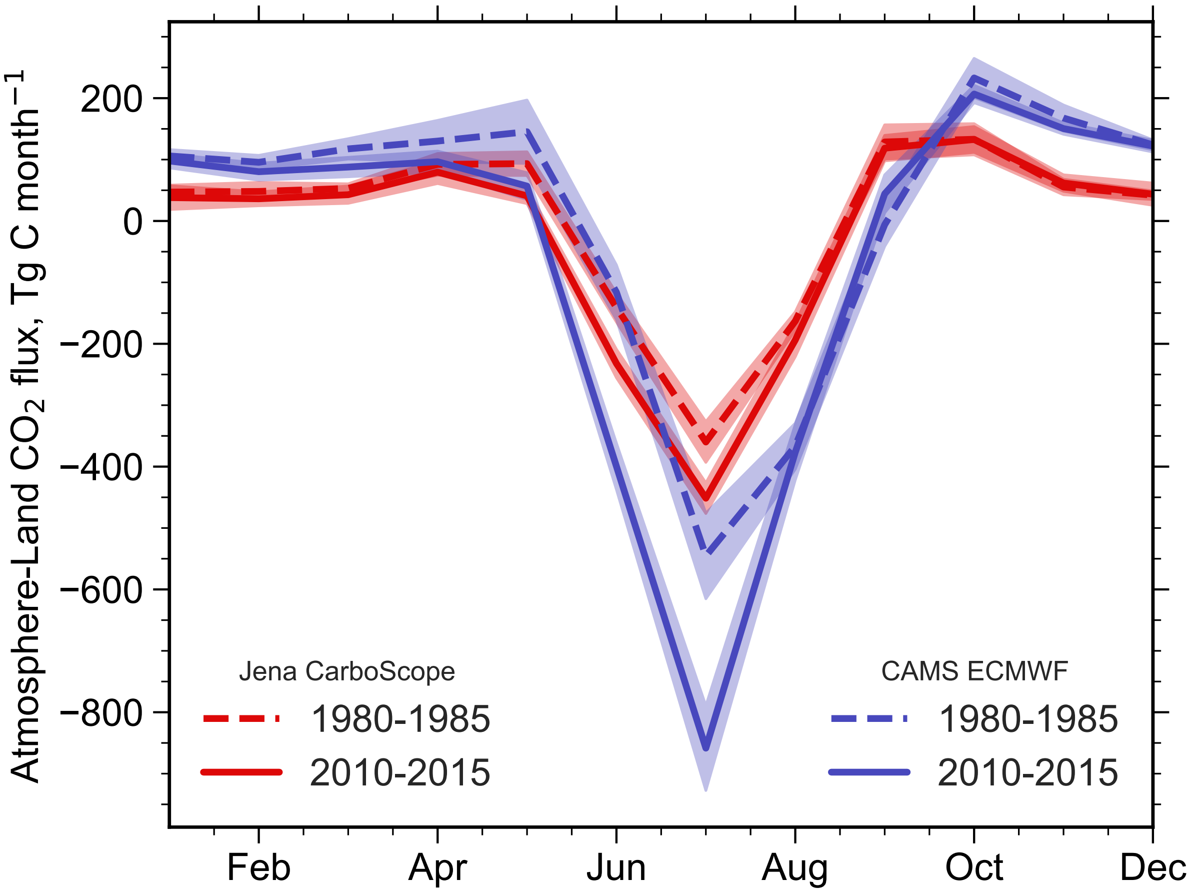

Supplementary Figure 5 | Atmospheric CO_2 inversions suggest strong increase of NHL land carbon sink. Seasonal cycle of land-atmospheric CO_2 exchange estimated by two inversion procedures, Jena CarboScope (red) and CAMS ECMWF (blue), for two time periods 1980-1985 (dashed) and 2010-2015 (solid). Shading indicates one standard deviation.

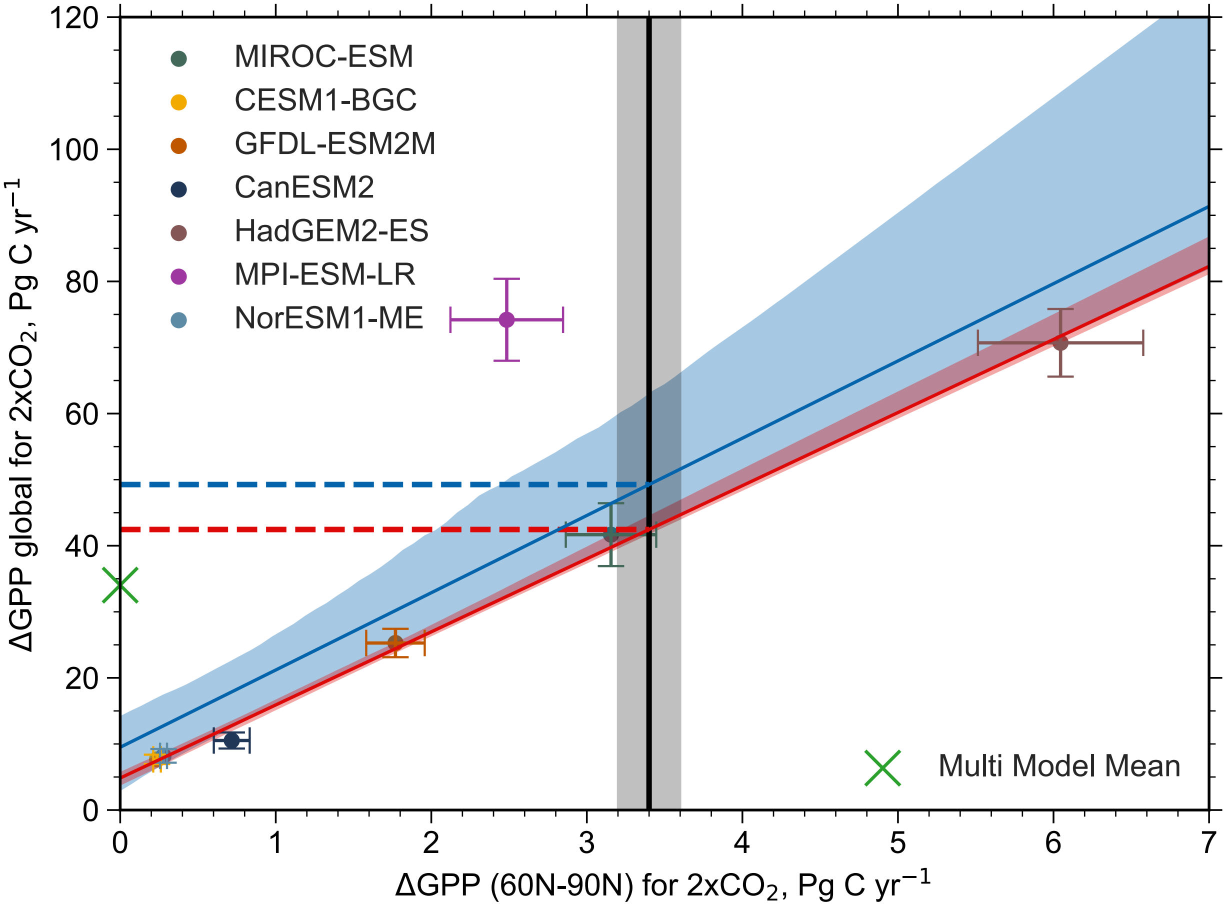

Supplementary Figure 6 | Strong linear relationship between global and NHL GPP increase in the CMIP5 ensemble. Comparison of NHL (*x*-axis) and global (*y*-axis) estimates of changes in annual mean GPP for doubling of pre-industrial CO_2 concentration. The markers show the individual model including error bars for one standard deviation. The black vertical line shows the observation-based Emergent Constraint estimate of NHL GPP increase with the gray shading indicating uncertainty (Methods). The blue line represents the best linear fit across the entire CMIP5 ensemble (blue shading denotes the 68% confidence interval), whereas the red line shows the best fit excluding the outlier MPI-ESM-LR (purple, the MPI-ESM-LR CMIP5 version is overly productive in the tropics due to almost absent water limitation). The dashed horizontal lines indicate the respective constraints on global GPP increase. Based on this result, also on global scale a substantial underestimation of photosynthetic carbon fixation is present – constrained estimate is 44% higher than multi-model mean indicated by green cross (56% excluding outlier).

Supplementary Table 1 | Summary data for Principal Component Analysis, LAI_{max} sensitivity estimation and GPP increase.

Model	Explained variance by ω	Offset to initial LAI_{max}, *a* (m^2 / m^2)	LAI_{max} sensitivity to ω, *b* (m^2 / m^2 unit ω)	Correlation coefficient	ΔGPP for 2xCO_2 (Pg C / yr)
MIROC-ESM	0.89	2.7 +/- 0.00075	0.049 +/- 0.0033	0.93	3.2 +/- 0.29
CESM1-BGC	0.83	0.44 +/- 0.00031	0.014 +/- 0.0014	0.86	0.24 +/- 0.025
GFDL-ESM2M	0.64	2.9 +/- 0.00062	0.022 +/- 0.0032	0.76	1.8 +/- 0.19
CanESM2	0.91	0.68 +/- 0.00023	0.013 +/- 0.001	0.91	0.72 +/- 0.12
HadGEM2-ES	0.94	1.5 +/- 0.00081	0.075 +/- 0.0035	0.97	6 +/- 0.53
MPI-ESM-LR	0.77	1.4 +/- 0.00038	0.028 +/- 0.0018	0.94	2.5 +/- 0.36
NorESM1-ME	0.84	0.21 +/- 0.00018	0.0088 +/- 0.00083	0.88	0.28 +/- 0.022
**Observations**	**0.9**	**1.8 +/- 0.0015**	**0.045 +/- 0.0064**	**0.78**	-

Supplementary Table 2 | Overview of CMIP5 models included in this study.

Models	MPI-ESM-LR	CanESM2	MIROC-ESM	NorESM1-ME	CESM1-BGC	GFDL-ESM2M	HadGEM2-ES
No. of PFTs	12	9	13	16	16	5	5
Land model	JSBACH	CTEM	SEIB-DGVM	CLM4	CLM4	LM3	TRIFFID
Land resolution	1.9° × 1.9°	2.8° × 2.8°	2.8° × 2.8°	2.5° × 1.9°	0.9° × 1.2°	2.5° × 2.5°	1.9° × 1.2°
Dynamic vegetation	Yes	No	Yes	No	No	Yes	Yes
Explicit nitrogen cycle	No	No	No	Yes	Yes	No	No
Reference	Raddatz et al. (2007)[51]; Reick et al. (2013)[52]	Arora et al. (2011)[53]	Watanabe et al. (2011)[54]	Bentsen et al. (2013)[55]	Lindsay et al. (2014)[56]	Dunne et al. (2012)[57,58]	Collins et al. (2011)[59]

Supplementary Table 3 | Correlation coefficients for the relations shown in Supplementary Fig. 3.

Models	MPI-ESM-LR	CanESM2	MIROC-ESM	NorESM1-ME	CESM1-BGC	GFDL-ESM2M	HadGEM2-ES
< 2xCO2	0.94	0.95	0.97	0.94	0.93	0.89	0.99
> 2xCO2 & < 3xCO2	0.78	0.83	0.89	0.77	0.82	0.067	0.96
> 3xCO2	0.51	0.67	0.63	0.27	0.62	0.12	0.78

Supplementary References:

51. Raddatz, T. J. *et al.* Will the tropical land biosphere dominate the climate–carbon cycle feedback during the twenty-first century? *Clim. Dyn.* **29**, 565–574 (2007).
52. Reick, C. H., Raddatz, T., Brovkin, V. & Gayler, V. Representation of natural and anthropogenic land cover change in MPI-ESM. *J. Adv. Model. Earth Syst.* **5**, 459–482 (2013).
53. Arora, V. K. *et al.* Carbon emission limits required to satisfy future representative concentration pathways of greenhouse gases. *Geophys. Res. Lett.* **38**, L05805 (2011).
54. Watanabe, S. *et al.* MIROC-ESM 2010: model description and basic results of CMIP5-20c3m experiments. *Geosci. Model Dev.* **4**, 845–872 (2011).
55. Bentsen, M. *et al.* The Norwegian Earth System Model, NorESM1-M – Part 1: Description and basic evaluation of the physical climate. *Geosci. Model Dev.* **6**, 687–720 (2013).
56. Lindsay, K. *et al.* Preindustrial-Control and Twentieth-Century Carbon Cycle Experiments with the Earth System Model CESM1(BGC). *J. Climate* **27**, 8981–9005 (2014).
57. Dunne, J. P. *et al.* GFDL's ESM2 Global Coupled Climate–Carbon Earth System Models. Part I: Physical Formulation and Baseline Simulation Characteristics. *J. Climate* **25**, 6646–6665 (2012).
58. Dunne, J. P. *et al.* GFDL's ESM2 Global Coupled Climate–Carbon Earth System Models. Part II: Carbon System Formulation and Baseline Simulation Characteristics. *J. Climate* **26**, 2247–2267 (2012).
59. Collins, W. J. *et al.* Development and evaluation of an Earth-System model – HadGEM2. *Geosci. Model Dev.* **4**, 1051–1075 (2011).
